# Periocular Desmoplastic Malignant Melanoma and Orbital Invasion With Uncontrollable Disease: A Case Report

**DOI:** 10.7759/cureus.60541

**Published:** 2024-05-18

**Authors:** Celia Ruiz-Arranz, Álvaro Bengoa-González, Marcelo Bencomo-Villegas, Mariña Veras-Lista, Enrique Mencía-Gutiérrez, Bianca-Maria Laslău, María-Dolores Lago-Llinás

**Affiliations:** 1 Department of Ophthalmology, 12 de Octubre Hospital, Complutense University, Madrid, ESP; 2 Department of Pathology, 12 de Octubre Hospital, Complutense University, Madrid, ESP; 3 Department of Ophthalmology, Clínica Imed, Sibiu, ROU; 4 Department of Ophthalmology, Oculoplastic Fellowship Program, 12 de Octubre Hospital, Madrid, ESP

**Keywords:** exenteration, orbital invasion, melanoma, desmoplastic, periocular, eyelid

## Abstract

Primary orbital melanoma and metastatic cutaneous melanoma of the orbit are extremely rare. Desmoplastic melanoma (DM) is an infrequent variant of melanoma that can extend from a superficial location into deep tissues by neurotropic mechanisms.

A 78-year-old male was referred to us with a periocular mixed malignant melanoma (spindle cell melanoma with desmoplastic reaction) in his left lower eyelid with uncontrollable disease (orbital and inferior orbital rim invasion) despite treatment. The surgical technique consisted of an extended orbital exenteration, maxillectomy, and ethmoidectomy, with a 2 cm macroscopic surgical margin. We performed a delayed socket reconstruction with a temporalis muscle flap using a transorbital approach.

The patient remained disease-free for 1.5 years with a good quality of life since exenteration surgery. At this time, he presented a recurrence in the area of the malar scar with a new orbital invasion, and finally, he died due to mediastinal, pleural, and pulmonary metastasis.

The treatment of a cutaneous melanoma arising in the periocular region is a challenging reconstructive problem and it may compromise the globe and visual function.

## Introduction

Cutaneous melanoma is one of the most aggressive skin cancer types and one of the main causes of death related to cancer due to its ability to metastasize. The incidence of cutaneous melanoma varies between countries due to differences in cutaneous phenotype and sun exposure, according to the Fitzpatrick skin type (I-VI) [1].

Desmoplastic melanoma (DM) is a rare subtype of spindle cell melanoma associated with a fibrotic stroma (desmoplastic), first described by Conley, Lattes, and Orr in 1971 [2]. This is a distinct subtype with its own prognosis factors and represents less than 4% of all cutaneous melanomas; DM has been further sub-classified into pure and mixed [3].

Patients with DM tend to present with more advanced local disease, but the overall prognosis is better with DM compared with other types of melanoma at the same stage.

We have found 13 cases of DM in the periocular region published in PubMed [3-13]. The first published in this location was by Sutula et al. in 1982 [4], and six of the cases had orbital invasion [4,6,7,9,10,12].

We herein present a case of a recurrent periocular DM with extension into orbital space and inferior maxillary bone. We describe the clinical, histological, and radiological characteristics and the surgical approach to remove the tumor.

## Case presentation

A 78-year-old Caucasian male presented with a medical history of arterial hypertension, dyslipidemia, and benign prostatic hyperplasia. He was a heavy smoker of 2 packs per day (82 packs-years) from 18 to 59 years old. He had a lesion in the middle third of his left lower eyelid, bordering the malar region and measuring 1.5 cm in diameter. It appeared two years earlier with slow growth initially but subsequently with rapid growth in the last two months. The lesion was oval, firm, well-circumscribed, discreetly pigmented, with pearled edges and ulcerated center. It presented repeated bleeding but did not adhere to deep planes or bone and had no globe involvement. There was no history of other skin lesions at that time. The Fitzpatrick skin type guide classification was IV (I-VI) according to the pigmentation and skin response to ultraviolet (UV) radiation. The initial clinical diagnosis was basal cell carcinoma.

The first surgical procedure was an excisional biopsy of the lesion, with security margins (1 cm) and delayed repair (slow Mohs) with a radial forearm free flap. Histopathological exam of the excised surgical specimen showed an ulcerated widely invasive melanoma (spindle cell and desmoplastic component), with a 12.2-mm Breslow thickness, 11 mitotic figures per mm^2^ (high tumor mutational load), and V level of Clark. It showed the BRAF wild-type gene, with negative real-time C-protein reactive for genetic detection of the V600E/K mutation. The excision margins were positive in depth and at the inner limit of the lesion. Immunohistochemical studies with antibodies for S100 protein and monoclonal antibody SOX10 confirmed the diagnosis of mixed melanoma (spindle cell and desmoplastic component, that is, DM). Perineural invasion was not demonstrated. Computed tomography (CT) scan did not show osseous infiltration.

**Figure 1:**
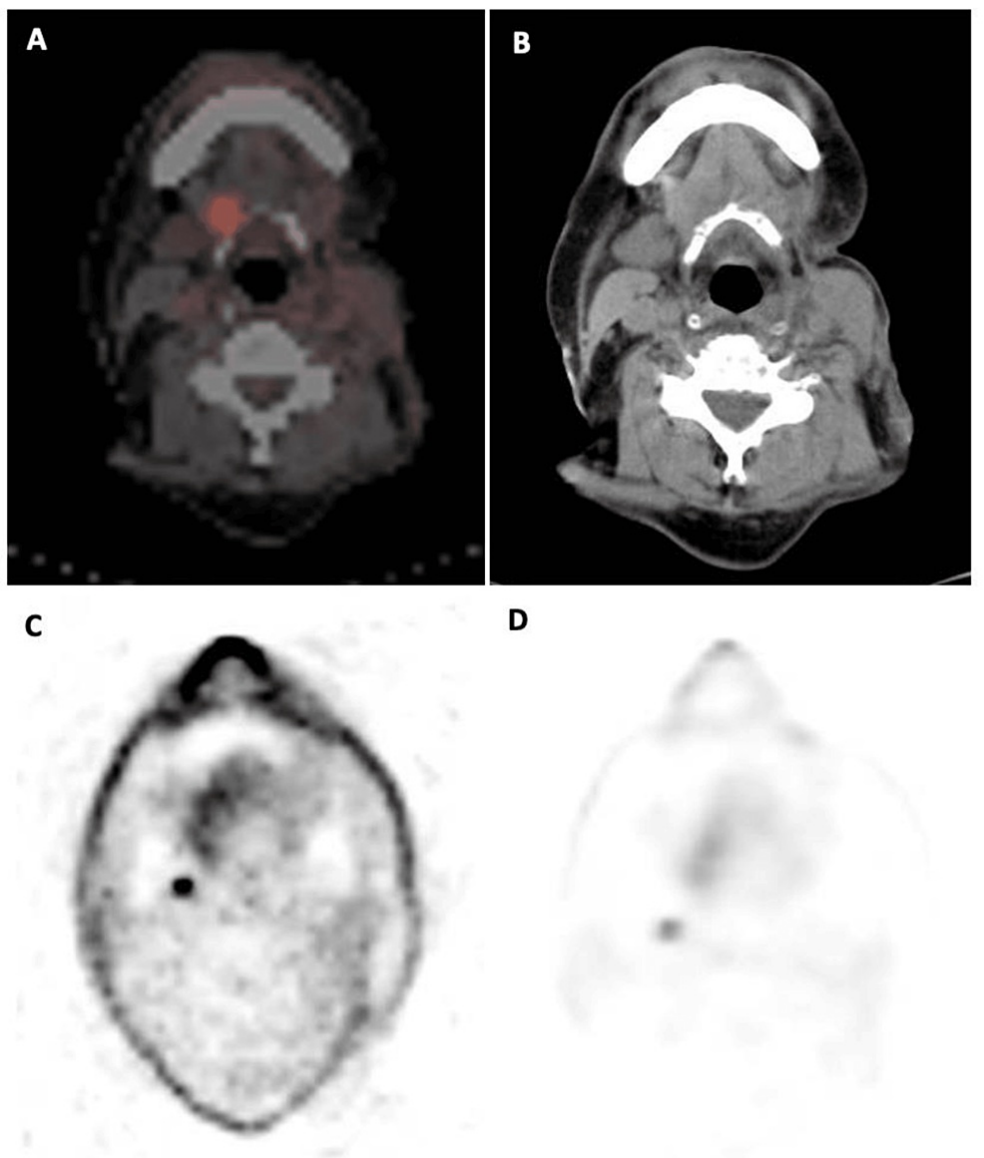
PET-CT showing an increased metabolic activity rate A and B: in left submandibular adenopathy; C and D: in left cervical adenopathy PET: Positron Emission Tomography. CT: Computed Tomography

A positron emission tomography (PET)-CT scan showed a left submandibular oval lymphadenopathy of 16 mm in its larger diameter and three more positive cervical nodes in group IIA. The scan also showed an increased metabolic activity rate of standardized uptake value (SUV) max of 4.99 in left submandibular adenopathy (Figure 1; A and B) and 3.74 SUV max in left cervical adenopathy (Figure 1; C and D).

The study was negative for the presence of lymph nodes, metastases, and other primary cancer in the rest of the body. The size extent of the primary tumor (T), involvement of regional lymph nodes (N), and the presence or absence of distant metastases (M) (TNM) according to the American Joint Committee on Cancer (AJCC) staging system for cutaneous melanoma resulted in stage IIIC and pT2bN2M0. Three weeks later, after the excisional biopsy, re-excision with a 2 cm margin in all directions was necessary due to positive surgical margins in addition to a resection of the inferior infraorbital nerve and collection of a bone sample from the inferior orbital rim. Besides, parotidectomy and left cervical lymphadenectomy (levels I, II, III, and IV) were performed. Immunotherapy (IT) was started at that time, a course of treatment with pembrolizumab based on the Eggermont et al. study [14], and with adjuvant modulated radiotherapy (RT): 50 Gray (Gy) on left I-III cervical areas and 64 Gy additionally on the II cervical area.

**Figure 2:**
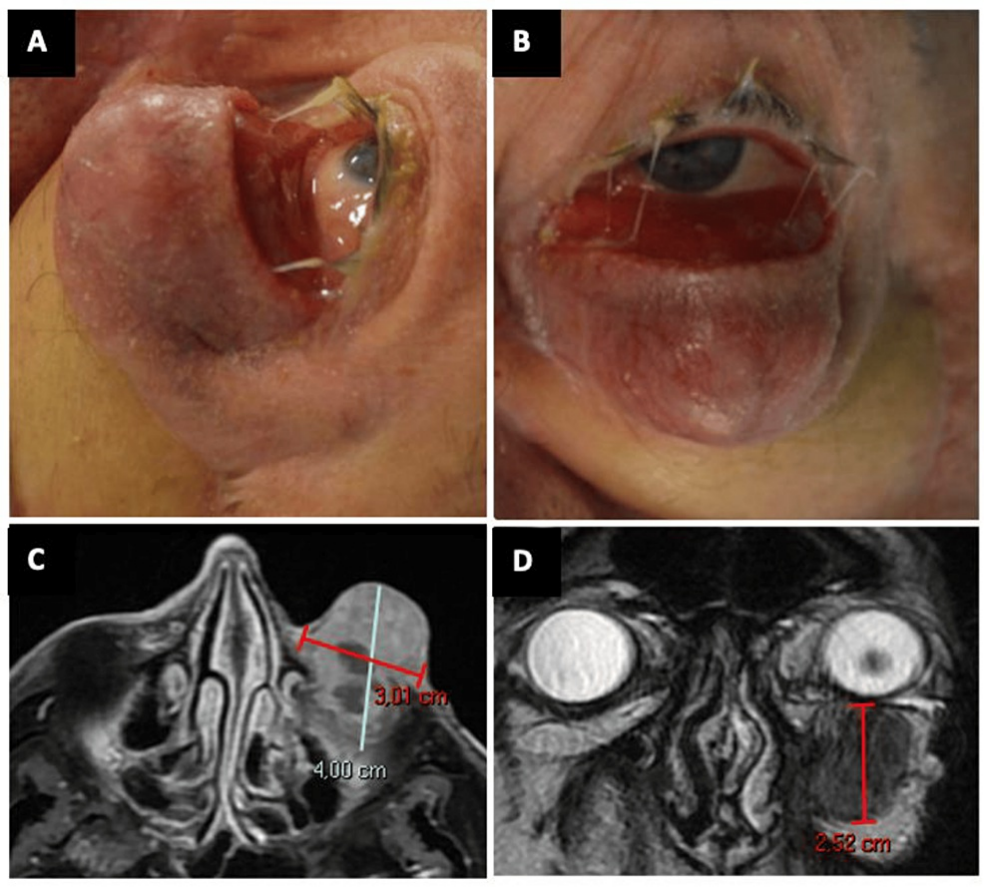
Clinical and radiological appearance of the local recurrence A, B. An amelanotic large mass involving the left conjunctiva and orbit; C, D. MRI of the left orbit showing orbital and maxillary involvement Axial (left) and coronal (right) T2-weighted spin-echo sequence shows a mass measuring 4.00*3.01*2.52 cm. MRI: Magnetic Resonance Imaging. Measures: anteroposterior*transverse*craniocaudal.

Due to immune-mediated toxicity, pembrolizumab was suspended and ipilimumab was started as the second-line IT regimen. Despite treatment, the tumor continued growing. At that time, the patient presented a large, firm, and sparsely pigmented mass into the conjunctiva with anterior orbital invasion, with a vertical displacement of the globe due to the tumor location (Figure 2; A and B), confirmed by magnetic resonance imaging (MRI) (Figure 2; C and D).

**Figure 3:**
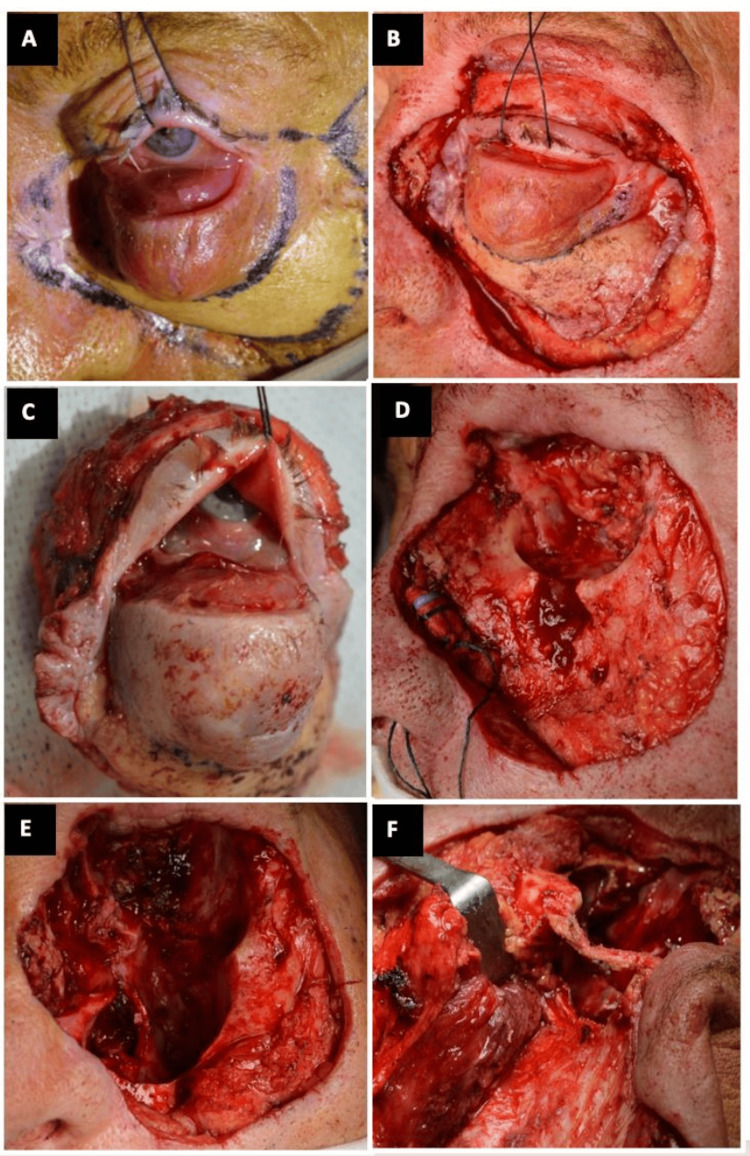
Extended total orbital exenteration, with maxillectomy and ethmoidectomy A. Surgical 2-cm security margins and temporalis flap design; B. Traction suture to perform exenteration and preserve superior eyelid skin; C. Total orbital exenteration piece, soft tissues, in one block; D. Bone appearance, with tumor infiltration of the left maxillary sinus; E and F. Socket appearance after soft tissue and bone removal (maxillectomy and ethmoidectomy) and bony window through the posterior lateral orbital wall, respecting the orbital rim. Both using the Sonopet® ultrasonic device

The surgical technique consisted of an extended total orbital exenteration (Figure 3; A-D) with maxillectomy and ethmoidectomy en bloc, using an ultrasonic bone curette device (Sonopet®; Stryker Corporation, Kalamazoo, Michigan, US) for the orbitotomy, creating the bony window through the posterior lateral orbital wall (Figure 3; E and F).

**Figure 4:**
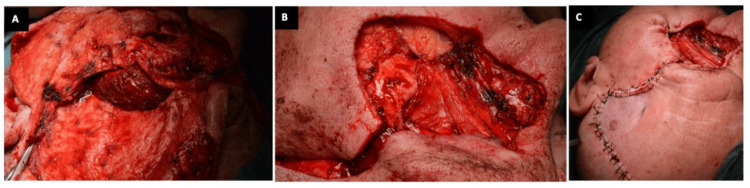
Intraoperative photos illustrating the surgical technique for socket reconstruction with a temporal muscle flap after total extended orbital exenteration A and B. The posterior 2/3rd temporalis muscle pedicle flap transferred into the orbit exenterated socket and into the maxillary sinus through an ostium created in the lateral orbital wall; C. Final image. The resulting defect was later covered with an ALT flap and a skin graft in a subsequent surgery ALT: Anterolateral Thigh

The reconstruction of both orbital and maxillary regions was delayed once tumor-free histopathological margins were ensured, and it was made by means of an ipsilateral temporal muscle flap with a single-stage transorbital approach, in which the muscle fibers enter the orbit through an ostium created in the lateral orbital wall, leaving the orbital rim intact (Figure 4A). The temporalis muscle flap was sutured to the surrounding tissue of the periorbital region (Figure 4B), filling the orbit socket and maxillary sinus cavity until final reconstruction was carried out three weeks later by using an anterolateral thigh muscle flap and a partial skin graft (Figure 4C). No titanium meshes were used in the reconstruction so as not to interfere with subsequent therapies if needed.

**Figure 5:**
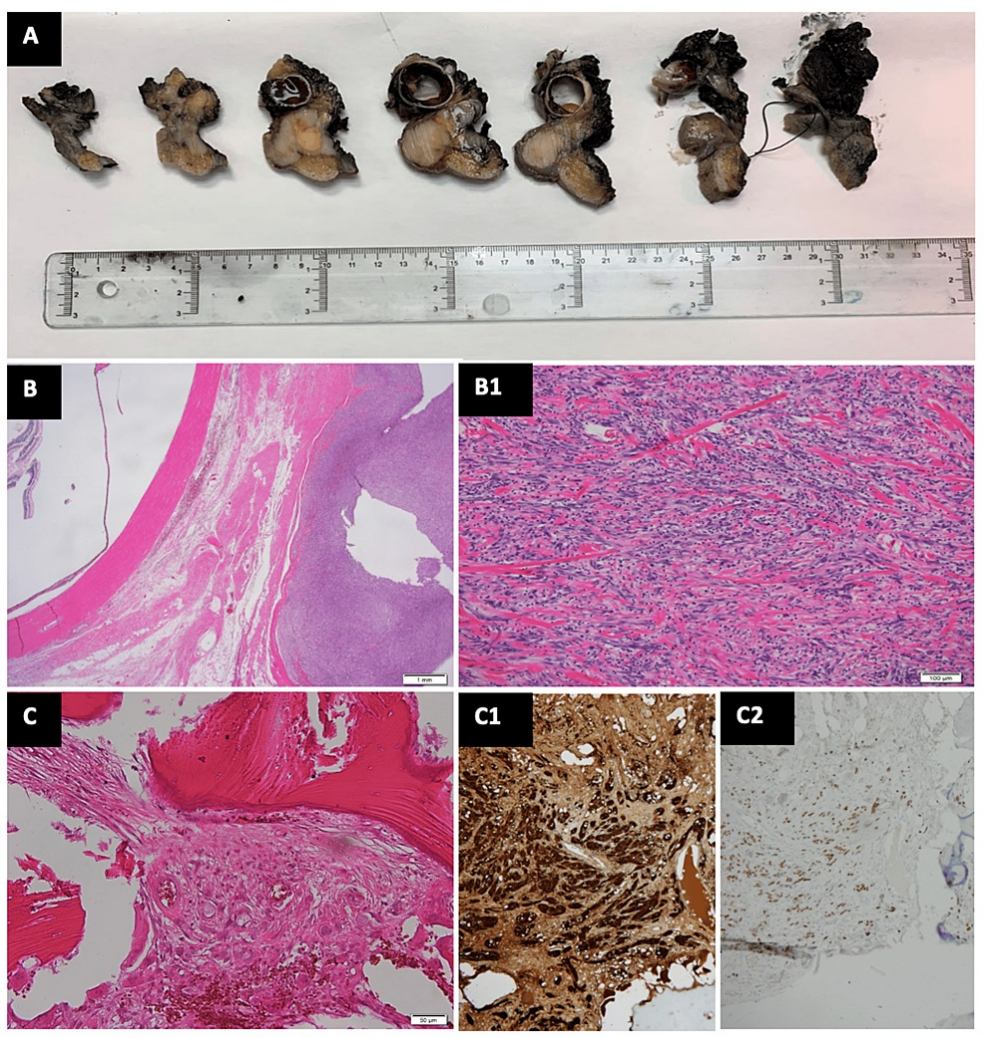
Histological evaluation of the excised surgical specimen A. Macroscopic tissue blocks for pathological study. B. Tumor that occupies the entire dermis and subcutaneous tissue, with consumption of the epidermis composed of epithelioid cells with marked cellular pleomorphism, prominent cytological atypia, and abundant mitotic figures (8 per square millimeter). The tumor occupies the soft tissues of the orbital region, remaining close to the eyeball (hematoxylin-eosin, x12.5). B1. Tumor cells interspersed with fibroepithelial cells and thick collagen bundles with a myxoid background are observed at the tumor margins (hematoxylin-eosin, x100). C. Bone tissue of the orbital floor with focal infiltration of melanoma (hematoxylin-eosin, x200). C1 and C2. Immunohistochemical studies with antibodies for S100 and SOX10, positive in tumor cells (C1, x200) and negative in bone tissue (C2, x100).

The macroscopic examination showed a tumor that occupied the soft tissues of the orbital region, remaining close to the globe (Figure 5; A). Histopathologic evaluation of the excised surgical specimen revealed a non-ulcerated spindle cell melanoma with desmoplastic reaction, with a 12.2-mm Breslow thickness, mitotic figures (8 per square millimeter) (Figure 5; B), and negative surgical margins in skin (Figure 5; B1), infraorbital and supraorbital nerves, nasal and maxillary mucosa, and bone orbital walls (Figure 5; C) and maxillary bone. Immunological studies with antibodies for S100 and SOX10 were positive again in tumor cells (Figure 5; C1, left side) and negative in bone tissue (Figure 5; C1, right side). The patient remained local and metastatic disease-free for 14 months until he developed a tumor recurrence on the nasal and maxillary area, as well as a great physical deterioration, due to metastatic disease and pulmonary progression (stage IVC).

**Figure 6:**
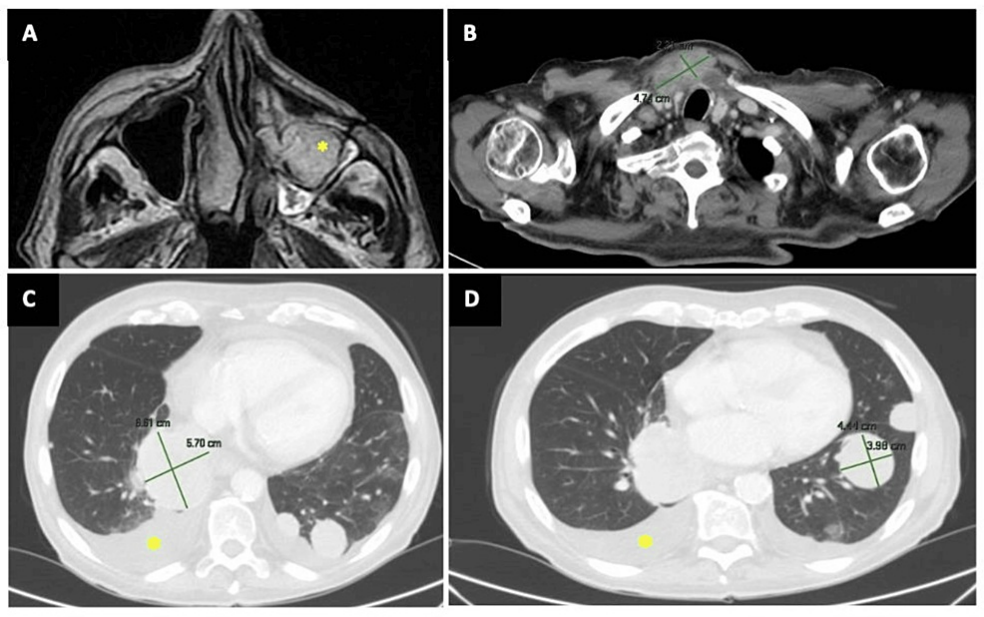
A. MRI shows an infiltrating mass in the maxillary sinus and nasal fossa (asterisk); B. Mediastinal lymphadenopathy measuring 4.7 x 2.2 cm in the jugular fossa; C and D. PET-CT image of lung metastases > 6 cm on the right side and > 4 cm on the left side, also pleural effusion (asterisk) PET: Positron Emission Tomography. CT: Computed Tomography. MRI: Magnetic Resonance Imaging

MRI showed malar osseous and sinus infiltration, as well as nasal cavity infiltration (Figure 6A). He presented multiple enlarged cervical lymph nodes (Figure 6B), and pulmonary, pleural, and mediastinal metastases (Figure 6; C and D).

**Figure 7:**
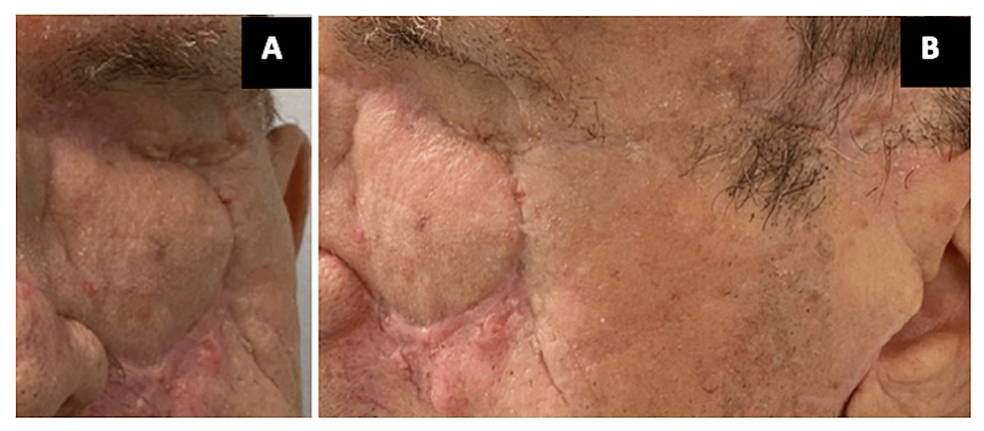
Follow-up at 12 months after exenteration, with good wound healing, flap integration, and good cosmetic appearance

He was treated with chemotherapy (QT) as the third-line treatment (dacarbazine). After four months, the patient refused any further treatment and was given palliative care, dying 10 days later. During the follow-up after the extended total orbital exenteration, he presented a good orbital filling effect and cosmetic acceptance with an improvement in his quality of life (Figure 7).

## Discussion

DM presents clinically as an insidious plaque, nodule, or scar, most of them amelanotic or poorly pigmented. Compared with spindle cell melanomas, DM appears later in life (average of 66 years old), in chronic sun-exposed areas (head and neck in 50% of the cases) [3], and most commonly in males [15,16].

The clinical and histopathological differential diagnosis should include hypertrophic scarring, amelanotic melanoma, basal cell carcinoma, squamous cell carcinoma, dermatofibroma, dermatofibrosarcoma, atypical fibroxanthoma, or tumors derived from peripheral nerve sheaths [5].

When the extent of desmoplasia is greater them 90%, the neoplasm is considered DM, whereas if the extent of desmoplasia is less than 90% but greater than 10%, the lesion is mixed DM [15]. Positive histopathological examination with S100 protein and SOX10 monoclonal antibody is very useful in this diagnosis [17]. Of the histopathologic subtypes, the pure subtype has a better prognosis in terms of disease-free survival and less potential to metastasize to lymph nodes. Our case was a mixed subtype, which courses with a worse prognosis [15,17].

DM presents local invasion with an estimated recurrence rate between 3.2% and 14% and is associated with neurotropism [16,18], although perineural invasion could not be demonstrated in our case. DM has a higher risk for local recurrence but a lower risk for lymphatic spread to lymph nodes than other subtypes of cutaneous melanoma [15,16,18]. Our patient, with pT2N2M0, IIIC stage at the time of diagnosis, was at high risk of developing nodal and systemic metastasis. At this stage, a 5-year survival rate of 67%-89% is estimated [14]. Melanomas involving the eyelids with a stage of at least T2b have been associated with a higher risk of nodal and systemic metastases [19].

Selective sentinel lymph node biopsy (SLNB) has an important prognostic value in patients with DM since most of them present as local disease and these tumors have a greater mean thickness [18]. As of today, taking into account that pure DMs metastasize less than mixed ones, SLNB is recommended in mixed ones, but there is no consensus on its usefulness in pure ones, with practical recommendations varying depending on the institution [16,18,19].

According to current clinical guidelines, our patient showed a probability of a positive sentinel lymph node >10% so SLNB was recommended [18]. Considering this, the patient's status, his previous tumor history, and its availability in our center, we decided to perform a PET-CT instead. PET-CT imaging also allows us to show the tumoral hypermetabolism of the subcutaneous recurrence and the presence of lymph nodes and metastasis.

To reduce the risk of local recurrence of DM, excision of the lesion must be performed with a 2-cm surgical margin [16], which in the periocular area means in some cases putting the eyeball at risk. An exhaustive anatomopathological study should be carried out, and the reconstruction should be left for a second time once the free margins have been confirmed.

Adjuvant RT should be considered to reduce the risk of local recurrence and IT should be considered when there is metastatic disease or a high Breslow index at diagnosis.

Local excision with free margins is the first line of treatment for all primary cutaneous melanomas. Due to the locally aggressive behavior of the DM, excision with 2 cm margins is recommended whenever possible for advanced tumors [16]. Breslow >4 mm y V Index Clark (hypodermis or subcutaneous fat invasion) makes the 5-year survival rate about 50%. Specifically, in the periorbital area, obtaining these margins can mean the loss of the eyeball and visual function, as it happened in our patient. Surgical treatment should be the first choice to perform in these cases; however, postoperative tumor recurrence and metastasis are still regarded as treatment failures. An exhaustive anatomopathological study should be carried out, and the reconstruction should be left for a second time once free margins have been confirmed. However, although it is not clear, local adjuvant RT and systemic QT should be used to treat patients with relapse, invasion or adjacent tissues, and systemic metastases [15,16], above all when a satisfactory histological clearance (≥ 8 mm) cannot be achieved [19]. Anti-PD1 drugs (pembrolizumab) are indicated as the first line of adjuvant treatment in patients with stage II or III, unresectable, metastatic, or lymph node-positive disease, having demonstrated an increase in disease-free survival [20]. Anti-CTLA-4 drugs are used as a second-line treatment (ipilimumab), as they have lower efficacy than anti-PD1 ones and greater adverse effects. We used dacarbazine, an alkylating agent, as a third-line QT.

IT using anti-PD1 drugs has demonstrated its efficacy in the treatment of metastatic DM, especially in those DM with a high mutational load (induced by UV radiation), and its effectiveness has been proven as adjuvant therapy after excisional surgery in patients with stage III melanoma [14].

The low frequency of BRAF V600E/K mutation in DM, which is present in only 10% of the cases and neither did our case [18], makes the use of BRAF inhibitors limited to a small number of patients. In non-DM melanomas, the mutation is present in almost 50% [16].

There are 13 cases of DM in the periocular area described in the scientific literature (PubMed); six of them presented with orbital invasion during recurrence [3-13].

We have not found any published report with an extended total orbit exenteration and en bloc resection with maxillectomy and ethmoidectomy, as in our patient. Exenteration with a single-stage repair of the orbital socket is a promising reconstructive option for exenterated sockets. Unlike previous reports, we performed the reconstruction after histological free-margin confirmation. Exenteration confers a significant survival rate in advanced periocular malignancies, even in patients with uncontrollable systemic disease, or where the local disease is deemed incurable [17,21-23].

Owing to the tumor extension in our patient, we performed a challenging reconstruction, and we had to compartmentalize the orbit from the nasal cavity and maxillary sinus, which is very well done by using large muscle flaps.

The single-stage transorbital approach for temporalis muscle pedicle transfer appeared as a good technique with reasonably good anatomical and cosmetic success [22], and the use of the Sonopet® ultrasonic bone curette provides speed and good control and visualization and handling of the tissues in osseous lateral wall osteotomy [23]. The temporalis muscle flap provides adequate orbital volume restoration and it also helps in better skin graft uptake, socket health, and appearance [22].

Our patient survived a total period of 3.5 years from initial diagnosis and 1.5 years since exenteration surgery, in which no socket complications were observed. He remained with a very good orbital cosmetic appearance with optimum volume preservation without the need for a custom-designed orbital prosthesis.

## Conclusions

Nowadays, a total orbital exenteration is performed only as a life-saving procedure in certain malignancies or infections and the most important contemplation after total orbital exenteration is the reconstruction of the orbital socket. The temporalis muscle flap repair gives promising results because it allows faster healing of the socket. To reduce the risk of local recurrence of DM, excision of the lesion must be performed with a 2-cm surgical margin, which in the periocular area means, in some cases, putting the eyeball at risk. An exhaustive anatomopathological study should be carried out, and reconstruction should be delayed until confirming negative tissue margins. Consider adjuvant RT to reduce the risk of local recurrence and IT when there is a metastatic disease or a high Breslow index at diagnosis. Despite the disfiguration that is inherent to this procedure, the aesthetic results are considered satisfying in most patients.
